# The Role of Corticosteroids in the Treatment of Pain in Cancer Patients

**DOI:** 10.1007/s11916-012-0273-z

**Published:** 2012-05-29

**Authors:** Wojciech Leppert, Tomasz Buss

**Affiliations:** 1 Department of Palliative Medicine, Poznan University of Medical Sciences, Osiedle Rusa 25 A, 61-245 Poznan, Poland; 2 Department of Palliative Medicine, Medical University of Gdańsk, Gdańsk, Poland

**Keywords:** Adjuvant analgesics, Cancer, Corticosteroids, Opioids, Pain

## Abstract

Pain is one of the most frequent and most distressing symptoms in the course of cancer. The management of pain in cancer patients is based on the concept of the World Health Organization (WHO) analgesic ladder and was recently updated with the EAPC (European Association for Palliative Care) recommendations. Cancer pain may be relieved effectively with opioids administered alone or in combination with adjuvant analgesics. Corticosteroids are commonly used adjuvant analgesics and play an important role in neuropathic and bone pain treatment. However, in spite of the common use of corticosteroids, there is limited scientific evidence demonstrating their efficacy in cancer patients with pain. The use of corticosteroids in spinal cord compression, superior vena cava obstruction, raised intracranial pressure, and bowel obstruction is better established than in other nonspecific indications. This review aims to present the role of steroids in pain and management of other symptoms in cancer patients according to the available data, and discusses practical aspects of steroid use.

## Introduction

Pain is a vital problem in cancer as it occurs in 30–50 % patients in earlier stages and in 70–90 % of these patients with advanced disease. The management of this symptom is generally based on the concept of the World Health Organization (WHO) analgesic ladder. This three-step framework was published for the first time in 1986 by the WHO to promote rational use of analgesic medications in the treatment of cancer pain. Step I recommends nonopioid analgesics for mild pain. Step II specifies the use of weak opioids for moderate pain. Step III comprises the use of strong opioids for severe pain [1]. Recent cancer pain management guidelines in Europe are based on European Association for Palliative Care (EAPC) recommendations [2, 3••]. The knowledge of both basic types of pain (nociceptive and neuropathic) and their responsiveness to opioids is necessary to achieve good pain alleviation. Medical data demonstrate that complete pain relief is rarely achieved in cancer patients; nonetheless, it can be significantly reduced [4].

To achieve better pain relief, apart from the use of the analgesics recommended by the WHO ladder, an appropriate application of adjuvant analgesics (i.e., for neuropathic pain), supportive drugs (for the prevention and treatment of opioid adverse effects) [5], and nonpharmacological measures such as radiotherapy and invasive procedures (nerve blockades and neurolytic blocks) [6] should be considered and applied. Certain data confirm some of the generally known rules, which are presented below. In patients with bone pain, opioids may be combined with nonsteroidal anti-inflammatory drugs (NSAIDs) and bisphosphonates along with local or systemic radiotherapy [7]. Neuropathic pain apart from opioid analgesics may require antidepressants, anticonvulsants, and local anesthetics. In patients with very severe neuropathic pain, the combination of opioids and *N*-methyl-D-aspartate receptor (NMDA) antagonists, namely ketamine, is recommended [8]. Patients with visceral, colicky pain, especially in the course of bowel obstruction, should be treated with opioid analgesics and spasmolytics such as hyoscine butylbromide, hyoscine hydrobromide, or glycopyrrolate [9].

Steroids can reduce pain intensity by inhibiting prostaglandin synthesis and reducing vascular permeability. Their role in controlling cancer pain and other indications in the course of cancer and some practical aspects of steroid use will be discussed in this article. According to the WHO and EAPC pain management recommendations, an adjuvant pain medication should be considered at each step of the WHO analgesic ladder, especially when the anti-inflammatory effect is needed.

## Steroid Classification and Clinical Consequences

Endogenous steroids, depending on their nature, site of synthesis, and specific clinical features, can be divided into four groups: corticosteroids (glucocorticosteroids and mineralocorticosteroids), progestogens, androgens, and estrogens. Generally, steroids are produced in the adrenals and gonads, and some other tissues such as the bowel, liver, prostate, and nervous system (neurosteroids) may synthesize or metabolize them [10]. Corticosteroids have a wide range of actions. The principal sites of action and clinical consequences of corticosteroid excess are presented in Table 1. The knowledge of different corticosteroid properties is an important factor in the therapeutic decision making process. Table 2 compares the most often used glucocorticosteroids with respect to the anti-inflammatory properties and the potency of sodium retention and the hypothalamic-pituitary-adrenal (HPA) axis suppression.

**Table 1:** The principal sites of action of glucocorticoids in humans and some clinical consequences of its excess

Site of action	Consequences of glucocorticoid excess
Brain/CNS	Emotional liability, depression, insomnia, enhanced appetite, psychosis
Eye	Glaucoma
Endocrine system	↓LH, FSH, TSH release, ↓GH secretion
Gastrointestinal tract	Peptic ulcerations, fatty liver
Carbohydrate/lipid metabolism	Overall diabetogenic effect
Adipose tissue distribution	Promotes visceral obesity (cushingoid appearance)
Cardiovascular/renal	Salt and water retention, atherosclerosis, hypertension
Skin/muscle/connective tissue	Protein catabolism/collagen breakdown, skin thinning, muscular atrophy (myopathy), impaired wound healing, acne vulgaris
Bone and calcium metabolism	↓Bone formation, ↓bone mass, and osteoporosis
Growth and development	↓Linear growth
Immune system	Anti-inflammatory action, immunosuppression

*CNS* central nervous system, *LH* luteinizing hormone, *FSH* follicle-stimulating hormone, *TSH* thyroid-stimulating hormone, *GH* growth hormone

(Adapted and modified from Stewart [11])

**Table 2:** Comparison of relative biologic potencies of different synthetic steroids

Steroid	Salt retention	Anti-inflammatory effect	HPA axis suppression
Cortisol	1	1	1
Prednisolone	0.75	3	4
Methylprednisolone	0.5	6.2	4
Fludrocortisone	125	12	12
Dexamethasone	0	26	17

*HPA* hypothalamic-pituitary-adrenal

(Adapted from Stewart [11])

The anti-inflammatory effect and vascular permeability reduction are the clinical results of steroid use. Because the mineralocorticoid properties of steroids may lead to a higher risk of sodium excess, potassium loss, and water retention (in such cases, a concurrent supplementation of potassium should be considered), cortisone or hydrocortisone are rarely employed for long-term anti-inflammatory therapy (due to the highest sodium-retaining potency). The negative feedback of the HPA axis by endogenous and synthetic steroids is well established. It is a dose- and time-dependent process. Consequently, a sudden cessation of corticosteroid therapy may result in adrenal failure. Clinically significant HPA axis suppression is rare if a steroid is administered for less than 3 weeks. Such patients can withdraw from steroids suddenly, with no harmful effects [11]. There are some clinical situations (i.e., myopathy or dysphagia) when steroids have to be discontinued even if they have been used much longer.

Various syndromes may occur when the glucocorticoid is withdrawn or reduced. The first is relapse of the disease for which the steroids were prescribed. The second is a combination of nonspecific symptoms of steroid withdrawal syndrome (i.e., lethargy, depression, anorexia, nausea, myalgia, or arthralgia). This syndrome must be distinguished from the suppressed HPA axis or relapse of the underlying disease. The third syndrome is acute adrenal insufficiency, which could be precipitated by surgery, intercurrent disease, or stress and may result in a hemodynamic collapse [12]. Clinicians are often unsure how to safely reduce the dose of steroids. The most suitable method of tapering has not been established as yet. After the glucocorticoid withdrawal, the hypothalamic and pituitary functions recover first, followed by the adrenocortical function. Full recovery of adrenal function can take months, or even up to a year [13], especially after prolonged steroid treatment with high doses. A possible scheme of steroid discontinuation is presented in Table 3.

**Table 3:** The proposal schedule of glucocorticoid withdrawal when used over 3 weeks

Prednisone or equivalent daily dose	Proposal schedule of tapering
≥ 7.5 mg	Reduce rapidly, e.g. 2.5 mg every 3–4 days
	then
5–7.5 mg	Reduce by 1 mg every 2–4 weeks
	then
< 5 mg	Reduce by 1 mg every 2–4 weeks

(Adapted and modified from Livanou et al. [13])

## Corticosteroids and Pain Management

The process of pain origination is called nociception. It consists of four stages: transduction (in peripheral nociceptors), transmission (via neurons), modulation, and pain perception. The possible role of steroids on every step of nociception has been raised even though the exact mechanisms remain unclear. The variety of proinflammatory cytokines synthesized or/and released during tissue injury are responsible for peripheral sensitization. This process leads to an increase in pain perception. The reduction in inflammation involved in this process decreases nociceptors activation, and thus, can diminish pain intensity. A decrease in pathological electrical activity of damaged neurons is also suggested [14]. The anti-inflammatory effect of glucocorticosteroids results from their ability to inhibit the expression of collagenase (the key enzyme involved in tissue degeneration during inflammatory mechanisms), reduce pro-inflammatory cytokines, and stimulate the synthesis of lipocortin (blocks the production of eicosanoids) [15].

As a consequence, corticosteroids are considered to be the most effective strategy against inflammatory pain. Steroids also have an anti-swelling effect. The reduction of peritumoral edema by the shrinkage of tumor in response to steroid therapy may lead to the improvement in analgesia in brain metastases [16] and spinal cord compression [17]. The modulation of neuroimmune interactions by corticosteroids and the decrease of spontaneous discharge in an injured nerve may reduce neuropathic pain. The neurosteroids produced in the central nervous system (CNS) or peripheral nervous system (PNS) modulate the γ-aminobutyric acid (GABA), NMDA, and P2X (for adenosine triphosphate or biphosphate) receptors, which all play a crucial role in the regulation of pain [18].

The receptors of various steroid hormones are expressed in several neural structures, allowing steroids to control the development, growth, maturation, differentiation, and plasticity of the CNS and PNS. By modulating neural activity and plasticity, the steroids are suspected to play an important role in pain sensation. Sex steroids (androgens and estrogens) are key factors accounting for the gender difference in pain and analgesia. Androgens, particularly testosterone, exert an analgesic effect in humans while estrogens were found to have both the hyperalgesic and analgesic effects, depending on the experimental conditions [19, 20]. Sex steroids in a rat model modulate the antinociceptive responses to opioids through the control of κ- and δ-opioid receptors in the spinal cord [21].

The most often prescribed corticosteroid for pain treatment is dexamethasone. Long-acting dexamethasone causes less fluid retention than other steroids because it has less mineralocorticoid effect. However, betamethasone (equipotent to dexamethasone), prednisone, and prednisolone also may be used [22]. Dexamethasone is metabolized by the hepatic enzyme CYP3A4 [23], and like other drugs metabolized in this way, it has numerous potential interactions. It may affect the metabolism of carbamazepine, tricyclic antidepressants, venlafaxine, dextromethorphan, and, to less extent, methadone. The effect of dexamethasone may be increased by CYP3A4 inhibitors and weakened by CYP3A4 inducers.

Corticosteroids are commonly prescribed in cancer patients for a variety of symptoms [24, 25]. These include some specific and non-specific indications. There is evidence for the use of corticosteroids for specific indications such as raised intracranial pressure, spinal cord compression, superior vena cava obstruction, and bowel obstruction. However, there has been little evidence for this in the literature with regard to hypercalcemia and nonspecific indications such as pain, nausea and vomiting, fatigue, cancer-induced anorexia-cachexia, depressed mood, or poor general well-being and dyspnea [26]. The recommended dosing of corticosteroids in different clinical situations is presented in Table 4.

**Table 4:** Corticosteroid doses for common indications in cancer patients

Clinical indication	Recommended dose, *mg*
Specific	
Raised intracranial pressure	8–16 mg dexamethasone daily
Spinal cord compression	16–32 mg dexamethasone daily (8–16 mg b.i.d.)
Superior vena cava obstruction	16–24 mg dexamethasone daily (8 mg b.i.d or t.i.d.)
Bowel obstruction	8–16 mg dexamethasone daily
Nonspecific	
Anorexia	4 mg dexamethasone; 10–20 mg prednisolone
Nausea and vomiting	4–8 mg dexamethasone
Bone and neuropathic pain	4–8 mg dexamethasone

(Adapted and modified from Exton [26])

## Adverse Effects

Corticosteroids display short- and long-term toxicities. The immediate adverse effects include immunosuppression, which may manifest as candidosis, hyperglycemia, and psychiatric disorders. Long-term effects comprise myopathy, peptic ulceration, osteoporosis, and Cushing’s syndrome [14]. Therefore, due to a wide range of adverse effects, the lowest effective dose should be used. Additionally, other medications to counteract steroid side effects can be prescribed. The combination of NSAIDs and steroids increases the risk of gastric bleeding 15-fold; therefore, it should be avoided or gastroprotective drugs ought to be administered concurrently [27]. Bisphosphonates should be considered in the case of elderly patients and patients at risk of osteoporosis, especially when chronic steroid therapy is intended. Due to the diabetogenic effect of steroids, regular measurements of glucose levels are needed in these patients as well. All patients should be reviewed regularly to ensure that the treatment benefits outweigh the risks [7].

In an uncontrolled study, oral candidosis and proximal myopathy were the most frequent adverse effects attributable to steroid therapy. The most common reason for discontinuation of dexamethasone treatment was death or general deterioration. A total of four (4 %) out of the 106 treated patients discontinued the treatment due to adverse effects such as restlessness, sleep disturbance, dyspepsia, and skin rash [24]. In another study, 181 (31 %) out of 582 cancer patients experienced troublesome adverse effects. The most common adverse effects were moon face (43 %), myopathy/muscle weakness (34 %), skin purpura (31 %), oral candidosis (28 %), and aggravated or triggered diabetes mellitus (17 %) [22]. Among 88 patients with brain metastases, the most frequently documented adverse effects of steroids were increased appetite (32 %), proximal muscle weakness (28 %), and insomnia (21 %). Only 25 % of treated patients did not experience steroid-induced adverse effects [28].

A comparison of prednisolone with dexamethasone showed that adverse effects of both drugs were similar, although more psychological changes (*P* < 0.02) and hyperactivity (*P* < 0.05) were observed in patients treated with dexamethasone [29]. When comparing betamethasone with prednisolone, 33 % and 20 % of cancer patients, respectively, were assessed as having troublesome adverse effects. Gastroprotectors were prescribed by 27 % of doctors to 75–100 % of their patients treated with corticosteroids, and 65 % of physicians prescribed these drugs when patients received corticosteroids and NSAIDs concurrently [22]. Although hiccup is not a very frequent adverse effect of dexamethasone, in a recent case series, Kang et al. [30•] presented dexamethasone-induced hiccup that was easily resolved by switching to an equipotent dosage of either prednisolone or methylprednisolone.

Dexamethasone is often administered subcutaneously. It is alkaline, so it is highly likely to be incompatible with acidic solutions. If dexamethasone is to be mixed with other drugs, as much diluents as possible should be added before the addition of dexamethasone. Dexamethasone is incompatible in mixtures with haloperidol, midazolam, promethazine, and levomepromazine. Glycopyrronium chemically interacts with dexamethasone, but no precipitate forms; therefore, this combination should be avoided. Dexamethasone is compatible in mixtures with morphine, diamorphine, oxycodone, fentanyl, alfentanil, hydromorphone, dihydrocodeine, tramadol, hyoscine butylbromide, hyoscine hydrobromide, metoclopramide, and ondansetron [31].

## Overview of Clinical Studies of Corticosteroids in Cancer Patients

There is little recent evidence for the effectiveness and toxicity of corticosteroids in cancer. Some uncontrolled studies reported benefits of corticosteroids on pain and symptom control in these patients. The issue of an appropriate steroid treatment is discussed in the literature, and many questions regarding this problem have been raised (What are the exact indications for corticosteroid use? When should the steroid treatment be started? How long are steroids effective in symptom management, including pain? What is the most effective steroid dose? What are the most common adverse effects in cancer patients? Do we prescribe steroids too often in palliative care?). The studies presented below attempted to answer these important queries.

Nauck et al. [32], in a multicenter study carried out in 55 palliative care units in Germany and one in Austria and Switzerland, established that corticosteroids were one of the commonly administered drugs and were administered to 33 % of those patients. Corticosteroids were prescribed to 17.8 % of patients on admission and 32.4 % of patients during the stay at inpatient units. Younger patients were treated more often with corticosteroids [32]. In a more recent study conducted among 406 patients with advanced cancer who were consulted by a palliative care team in an outpatient clinic, corticosteroids were administered to about 25 % of patients [33]. A retrospective observation of corticosteroid use at the end of life in hospice patients conducted by Gannon et al. [25] revealed and confirmed high prevalence of corticoid use. A total of 51 % of 178 patients received corticosteroids, which were continued until death in 53 %. Only 2 % were switched from oral to parenteral corticosteroids. The main indications included treatment for raised intracranial pressure and to give a “boost.” The foremost reason for discontinuation of steroids was loss of the oral route.

According to the previously presented data, corticosteroids were suspected to improve pain control. Bruera et al. [34] conducted a randomized, double-blind, crossover study in 40 patients with advanced cancer. Oral methylprednisolone, 16 mg twice daily, or placebo was administered for 5 days. Then the patients were crossed over to the alternate treatment after 3 days of washout period. All the patients were then given methylprednisolone for 20 days. A total of 28 patients had pain of either bone, visceral, or neuropathic origin. Visual analogue scale scores for pain and analgesic intake were lower in the case of the methylprednisolone treatment in all types of pain. The benefit disappeared after day 33 in one third of the patients.

In a Swedish survey, out of a total of 147 patients with pain, 50 (34 %) had very good effect, 71 (48 %) had some effect, 4 (3 %) had no effect, and the effect of the treatment with corticosteroids could not be assessed in 22 patients (15 %). The positive response came within a week and lasted for more than 4 weeks. Of these patients, 81 % used corticosteroids for more than 4 weeks and 90 % were on betamethasone, with daily doses less than 3 mg in most of the patients [22].

Della Cuna et al. [35] reported a randomized double-blind study conducted among 43 patients with advanced cancer who were given either methylprednisolone, 125 mg intravenously daily, or placebo for 8 weeks. Visual analogue scale scores for pain improved in the methylprednisolone-treated group. However, the follow-up study conducted among 173 women did not confirm analgesic effects of methylprednisolone [36]. Tannock et al. [37] reported an uncontrolled study of oral prednisone, 7.5–10 mg daily, in 37 patients with metastatic prostate cancer. Bone pain was relieved in 14 patients. The response appeared to correlate with a decreased level of androgens [37].

Greenberg et al. [38] reported an uncontrolled study of 83 patients with spinal cord compression and pain who received dexamethasone, 100 mg intravenously, followed by 24 mg orally four times daily tapered over the period of 2 weeks. The patients were treated concurrently with radiotherapy. Pain relief was observed in 82 % of patients, in some patients before the commencement of radiotherapy. In an uncontrolled study, Kozin et al. [39] demonstrated pain relief in 63 % of 64 patients with reflex sympathetic dystrophy. The initial dose of dexamethasone was 60–80 mg daily with subsequent taper. Positive scintigraphy predicted a positive response to the treatment.

Sturdza et al. [28] reported the use of steroids among oncologists and palliative care physicians in the management of patients with brain metastases. Out of 38 physicians approached, 34 responded to this electronic survey; 45 % routinely used dexamethasone, 4 mg four times daily (16 mg daily). The others determined the dose of steroid according to the presence or absence of neurological symptoms. The dose was tapered over 4 weeks following completion of the whole brain radiotherapy by 60 % of the physicians.

Hanks et al. [29] observed that dexamethasone showed tendency for better results than prednisolone in patients with pain due to compression of the nerve. A total of 16 out of 34 patients responded to the treatment: 8 (38 %) out of 21 patients treated with prednisolone and 8 (62 %) out of 13 patients treated with dexamethasone. However, this trend could have been associated with relatively higher doses of dexamethasone (4 mg daily, *n* = 7; 8 mg, *n* = 4, 16 mg, *n* = 2) compared with the doses of prednisolone (30 mg daily, *n* = 10; 20 mg or less, *n* = 11) [29].

Several case reports demonstrated the effectiveness of corticosteroids in bone pain treatment. Arkel et al. [40] demonstrated effective use of corticosteroids in intractable bone pain in the course of hairy cell leukemia [40]. Vyvey [41•] depicted a patient with a pancreatic carcinoid with metastases to the liver and spine and mixed bone and neuropathic pain. The patient suffered from moderate pain despite hydromorphone administration. The patient was successfully treated with dexamethasone, 8 mg per day orally, which was subsequently reduced after a few days to the dose of 4 mg per day orally for 3 months.

Corticosteroids are commonly used for quality of life and symptom control improvement. Hardy et al. [24] conducted an uncontrolled study among 106 consecutive patients who started dexamethasone treatment according to the established prescription policy. The patients were surveyed each week to document the indications for use, beneficial effects, toxicity incurred and the reason for discontinuation of the treatment. All patients were diagnosed with advanced cancer and the median survival time was 40.5 days (range: 1–398 days) from the start of dexamethasone treatment. Three or more assessments were completed by 57 % of patients. The most common specific indications for treatment with dexamethasone were spinal cord compression, cerebral metastases, lymphangitis carcinomatosa, and bowel obstruction. The most common nonspecific indications comprised anorexia, nausea, low mood, pain, and vomiting. The median duration of steroid use was 21.5 days (range: 1–89 days). Symptom scores improved during the therapy compared with baseline in most patients for anorexia, nausea, pain, low mood, vomiting, and weakness, but not with respect to dyspnea and poor mobility.

Beneficial effects of steroid use on appetite, vomiting, general well-being, and social interaction in the Dellacuna et al. [33] study were noticed. In doctors’ assessment, the positive effect of steroids was seen by 71 % of physicians in poor well-being, 53 % in appetite loss, 45 % in nausea, and 40 % in fatigue. Moertel et al. [42] reported a significant improvement in appetite in a randomized, double-blind, placebo-controlled study of dexamethasone in 116 patients with advanced gastrointestinal cancer after 4 weeks, although weight did not increase. Improvements in depression, appetite, and food consumption also were observed. Strength was insignificantly improved, and performance status and survival were not different form control.

The problem of appropriate steroid dose and its efficacy is raised in the literature and clinical practice. In a randomized double-blind trial Vecht et al. [43] compared initial dexamethasone dose of 10 mg versus 100 mg in 37 patients with spinal cord compression who then received radiotherapy. No significant differences were found between the patient groups with respect to pain relief.

Lundström and Fürst [22] asserted that betamethasone (equipotent to dexamethasone) was the most commonly prescribed drug followed by prednisolone. Dexamethasone was used only by a few physicians. The dose for the treatment of anorexia, fatigue, or low mood was betamethasone, 3.5 mg, or prednisolone, 17 mg. The mean starting doses for the treatment of nausea were 4.8 mg and 19 mg, respectively. The dose was tapered to maintenance dose if possible by 75 % of physicians; 83 % stated that more than 50 % of their patients had a positive effect of the treatment, and 97 % of the respondents stated that the positive effect came within 5 days.

Hanks et al. [29] reported an uncontrolled study examining the effects of prednisolone or dexamethasone in 218 (58 %) of a total 373 patients with advanced cancer admitted to the inpatient unit. The duration of treatment with corticosteroids varied from 1 day to almost 11 years; the median duration was between 4 and 8 weeks. Patients received either prednisolone (*n* = 121) or dexamethasone (*n* = 95) in starting doses of 10–30 mg and 4–16 mg daily, respectively. The maintenance daily doses varied considerably from 5–20 mg of prednisolone and from 0.5 mg on alternate days to 4 mg twice daily of dexamethasone. There was no difference in response rates between the two drugs.

The beginning and duration time of the clinical effect of corticosteroids is another unclear problem. In the Swedish study, the authors noted that positive effect of steroids lasted from 3 to 6 weeks. Of the questioned doctors, 83 % stated that more than 50 % of their patients had a positive effect from the treatment, and 97 % of the respondents observed the positive effect within 5 days [22].

## Conclusions

Corticosteroids have an established role in specific indications in cancer patients such as spinal cord compression, superior vena cava syndrome, brain metastases with raised intracranial pressure and bowel obstruction. Their role in non-specific indications is not well proved. Clinical practice and several studies suggest that corticosteroids may be effective in the treatment of bone and neuropathic pain, when administered along with opioids and with other adjuvant analgesics. The decrease in pain intensity is probably connected with both the anti-inflammatory and anti-swelling effects as well as modulation of neuroimmune interactions. The sex-steroid dependent differences in pain sensitivity were also found.

It seems that dexamethasone may be commonly used for cancer pain management due to its high potency, long duration of action and minimal mineralocorticoid effect. A proposed starting daily dose is 8 mg (orally, subcutaneously or intravenously) with subsequent adjustment to the analgesia achieved and adverse effects. The lowest effective dose should be used and patients must be followed up regularly to assess benefits and risks associated with the therapy. In the situation when the general condition of a patient deteriorates and the patient is no longer able to swallow medications that are taken orally, it is rather recommended not to stop the treatment abruptly but to continue dexamethasone administration by the subcutaneous route.

Patients with severe pain intensity of bone and neuropathic origin should also be considered for the use of analgesic ladder step 3 opioids (opioids for moderate to severe pain, strong opioids) without climbing up the analgesic ladder. Other co-analgesics appropriate for bone and neuropathic pain should also be considered. Future studies that would take patients’ quality of life into consideration and could establish the role of corticosteroids in pain and other symptom treatment, are urgently needed.
